# Dynamin regulates metaphase furrow formation and plasma membrane compartmentalization in the syncytial *Drosophila* embryo

**DOI:** 10.1242/bio.20149936

**Published:** 2015-02-06

**Authors:** Richa Rikhy, Manos Mavrakis, Jennifer Lippincott-Schwartz

**Affiliations:** 1 Cell Biology and Metabolism Program, NICHD, NIH, Building 18T, 101, 18 Library Drive, Bethesda, MD, USA.; 2 Institut de Biologie du Développement de Marseille, CNRS UMR7288, Aix-Marseille Université, 13288 Marseille, France.; * Present address: Indian Institute of Science Education and Research, Homi Bhabha Road, Pashan, Pune, 411008, India.

**Keywords:** Dynamin, Endocytosis, *Drosophila*, Polarity, Actin, Compartmentalization, Syncytium

## Abstract

The successive nuclear division cycles in the syncytial *Drosophila* embryo are accompanied by ingression and regression of plasma membrane furrows, which surround individual nuclei at the embryo periphery, playing a central role in embryo compartmentalization prior to cellularization. Here, we demonstrate that cell cycle changes in dynamin localization and activity at the plasma membrane (PM) regulate metaphase furrow formation and PM organization in the syncytial embryo. Dynamin was localized on short PM furrows during interphase, mediating endocytosis of PM components. Dynamin redistributed off ingressed PM furrows in metaphase, correlating with stabilized PM components and the associated actin regulatory machinery on long furrows. Acute inhibition of dynamin in the temperature sensitive *shibire* mutant embryo resulted in morphogenetic consequences in the syncytial division cycle. These included inhibition of metaphase furrow ingression, randomization of proteins normally polarized to intercap PM and disruption of the diffusion barrier separating PM domains above nuclei. Based on these findings, we propose that cell cycle changes in dynamin orchestrate recruitment of actin regulatory machinery for PM furrow dynamics during the early mitotic cycles in the *Drosophila* embryo.

## INTRODUCTION

Early *Drosophila* embryo development occurs in a syncytium where the nuclei divide without PM boundaries. Each syncytial division cycle involves successive rounds of PM furrow ingression and regression (Foe and Alberts, 1983; Karr and Alberts, 1986; Kellogg et al., 1989). The syncytial cycles serve important morphogenetic roles in the early *Drosophila* embryo. Deeply ingressed furrows (6–9 µm in length) in metaphase possibly keep mitotic spindles isolated so they remain bipolar, enabling proper segregation of daughter nuclei during mitosis (Afshar et al., 2000; Stevenson et al., 2002). Shortened furrows (1–3 µm) in interphase, found in the intercap region, surround individual nuclei, helping to position nuclei uniformly across the embryo cortex. Both interphase and metaphase furrows organize the embryo PM into discrete polarized units prior to cellularization (Frescas et al., 2006; Mavrakis et al., 2009). The polarized PM units have epithelial-like properties, including an apical domain residing above individual nuclei and a lateral domain that forms the furrow membrane between nuclei. Restricted diffusion of PM components across the furrow gives rise to barriers within the syncytial PM that may help shape morphogen gradients in the embryo (Mavrakis et al., 2009).

Furrow dynamics during the syncytial division cycles are controlled by rapid changes in cytoskeletal and PM activities. Furrow ingression in prophase is driven by actin redistribution from the cortical caps above nuclei to lateral actin rings around dividing nuclei, driving invagination of the PM into the embryo. Microtubules, together with numerous actin regulatory components (e.g., Diaphanous, Peanut and Anillin), direct actin re-arrangements during this process, with F-actin contractile machinery assembling along the lateral edges of the furrow canals (Adam et al., 2000; Afshar et al., 2000; Field et al., 2005; Rothwell et al., 1998; Royou et al., 2004). Furrow regression in anaphase, on the other hand, coincides with actin redistribution back to caps above nuclei leading to furrow shortening. Ongoing exocytosis and endocytosis help fuel the PM growth and resorption during furrowing (Cao et al., 2008; Riggs et al., 2003; Sokac and Wieschaus, 2008). Specifically, endocytosis is stimulated both during furrow extension from interphase to metaphase, and during furrow regression during telophase (Sokac and Wieschaus, 2008). At cellularization, endocytosis is restrained due to expression of *nullo*, an inhibitor of endocytic machinery. This stabilizes furrow components for deeper invagination during cellularization (Fabrowski et al., 2013; Postner and Wieschaus, 1994; Sokac and Wieschaus, 2008). Hence, both membrane trafficking and actin dynamics during early development are highly regulated to achieve specific morphogenetic effects. However, the mechanism by which endocytic and actin-based machinery cooperate in the furrow ingression/regression cycle is still unclear.

In this study, we explore the role of dynamin in organizing the actin remodelling proteins to mediate the furrow dynamics and compartmentalization during the early syncytial mitotic cycles in the *Drosophila* embryo. Dynamin is a well-known regulator of various forms of endocytosis, catalyzing membrane scission of clathrin-coated and noncoated vesicles from the PM (Mettlen et al., 2009). When dynamin fails to undergo GTP hydrolysis/binding, as in the temperature sensitive (ts) *shibire* (*shi*^ts^) mutant form of dynamin (Damke et al., 1995; Narayanan et al., 2005) recycling cargo is unable to undergo fission at the PM (Damke et al., 1994; Damke et al., 2001). Dynamin also influences the contractile activity of actomyosin encircling epithelial cells at their apical cell-cell junctions, resulting in apical constriction in cells expressing a form of dynamin unable to bind GTP (Chua et al., 2009; Mooren and Schafer, 2009). Here, we find that dynamin undergoes changes in its localization during the furrow cycle: specifically associating with the furrow PM during interphase and depleting from it at metaphase. Other molecules involved in PM polarity and actin dynamics are affected in dynamin mutant embryos. Expression of the *shi*^ts^ mutant form of dynamin resulted in developmental consequences in the embryo, including disrupted metaphase furrows and loss of PM compartmentalization across the embryo. Based on these results, we propose a model in which changes in dynamin localization and activity during the mitotic cycle help coordinate actin and PM remodeling to form metaphase furrows in the syncytial *Drosophila* embryo.

## MATERIALS AND METHODS

### Generation of transgenic flies

The wild-type (WT) dynamin cDNA clone was obtained from Toshi Kitamoto (University of Iowa, Iowa, USA). The cDNA was amplified with primers 5′-GCCGCTCGAGATGGATAGTTTAATTACAATTGTTAA and 5′-TCCCCCCGGGCACGACGCGATGGTAGCATTG containing the Xho1 and the Xma1 restriction site respectively and sub cloned into the pEGFP-N1 vector (Clontech). The *shi*^WT^-GFP fragment was amplified using the primers 5′-GGGGTACCATGGATAGTTTAATTACAATTGTT-3′ and 5′-ATTTGCGGCCGCTTACTTGTACAGCTCGTCCAT-3′ containing the restriction sites Kpn1 and Not1 respectively from the clones and subcloned into the pUASP vector. The *shi*^ts2^ mutation (G141S) was made by quick-change site directed mutagenesis using the primers 5′-AAGGTGGCCATTAGCGATCAACCGG-3′ and 5′-CCGGTTGATCGCTAATGGCCACCTT-3′. The Clathrin light chain GFP in the pUAST vector was obtained from Henry Chang (Purdue University, West Lafayette, USA). It was amplified and subcloned into the pUASP vector using primers 5′-ATAAGAATGCGGCCGCATGGTGAGCAAGGGCGAG-3′ and 5′-GCTCTAGACTAGGTGCTCTTCTGCACC-3′ containing the Not1 and Xba1 restriction sites respectively. The *shi*^WT/ts2^-GFP and Clc-GFP clones in the pUASP vector were sent for injection to Duke University or Best Gene. The transgenic flies obtained were genetically mapped to chromosomes using standard crosses. Meiotic recombinants were made with nanos-Gal4 and further recombinants were made with *shibire* mutants using standard genetic crosses.

The cortactin cDNA (LD29964) was obtained from *Drosophila* Genome Research Center (DGRC). The cDNA was amplified using the primers 5′-GGGGTACCATGTGGAAGGCAAGTGCCG-3′ and 5′-TCCCCCCGGGGTGAGTTCTGTCCCACCACC-3′ and digested with Kpn1 and Xma1 to subclone it in frame with the CeFP. The Cortactin-CeFP was then sub-cloned by PCR using the primers 5′-GGGGTACCATGTGGAAGGCAAGTGCCG-3′ and 5′-ATTTGCGGCCGCTTACTTGTACAGCTCGTCCAT-3′ and digesting with Kpn1 and Not1 into the pUASP vector.

*shi*^ts2^ mutant flies were recombined with Dyn^ts2/WT^-GFP, Cortactin-CeFP, Spider-GFP and Moesin-GFP using standard genetic crosses.

### Processing embryos for live imaging and image quantification

Live embryos were processed for imaging as described in Mavrakis et al., 2008. Briefly, flies containing fluorescent transgenes were crossed with *nanos*-Gal4 and the progeny were put in cages (Genesee Scientific, USA) containing apple juice agar plates. Flies were allowed to lay eggs for 1 hour at 25°C. Embryos were washed with water in an egg collection chamber and treated with 100% bleach for 1 min to remove the chorion. They were washed in water again, dried in the mesh by blotting against a filter paper and mounted in a Labtek chamber with the help of a brush. They were covered with phosphate buffered saline. Image quantification was done using Image J. The line tool was used to estimate the average fluorescence per unit area in the polygons in the syncytium or depict the fluorescence across the line in wild type and mutant embryos.

### Immunostaining

Embryos were collected in cages for 1 hr, aged to the required developmental stage (typically 1 hr for getting the syncytial divisions) and heat shocked at 32°C for 5 min in temperature shift experiments or heat shocked for 5 min followed by a period of recovery of 5 min in recovery experiments. They were dechorionated in 100% bleach for 1 min. Embryos were fixed in 1:1 ratio of 4% paraformaldehyde in 0.1 M Pipes, 1 mM MgCl_2_ and 1 mM EGTA or phosphate buffered saline with heptane for 15 min at room temperature. They were washed with heptane and subsequently devitellinized with methanol (for Spider-GFP, Anillin, Peanut, E-Cadherin, Patj, Rab5) or hand-devitellinized (for phalloidin, Diaphanous). Embryos were rehydrated in phosphate buffered saline with 0.3% Triton-X100 (PBT) and blocked with 2% BSA in PBT. For Tubulin immunostaining the embryos were fixed in 1:1 ratio of heptane and methanol for 30 sec, washed in methanol and rehydrated. Primary antibodies were diluted in PBT containing BSA (anti-GFP 1:1000; Diaphanous 1:500; Anillin 1:1000; Peanut 1:5; Patj 1:1000; Rab5 1:100) and secondary antibodies were diluted in PBT (Fluorescent secondary antibodies from Molecular Probes 1:400; phalloidin Texas-Red or phalloidin 633 1:20). To control for sources of variation between samples to be compared at the same time, control and mutant embryos were processed at the same time and imaged on the same day. The stage of the syncytial cycle was determined by the estimation of the length of the lateral domain of the plasma membrane as characterized previously in Mavrakis et al., 2009 and morphology of the nucleus or the morphology of the staining with Hoescht dye during immunostaining. Typically interphase furrows show actin caps and are 2 µm deep whereas metaphase furrows show actin rings and are 6–9 µm deep.

### Live Imaging

Live imaging and imaging of fixed embryos was carried out on the Zeiss Confocor or Meta 510 confocal laser-scanning microscope. Either single optical sections or Z stacks were acquired over time and movies represent a projection of the Z stacks. For temperature shift experiments, control and mutant embryos were imaged at the same temperature. The temperature was maintained with a circulating water stage connected to a water bath maintained at the desired temperature. In addition, a heater fan was also used to compensate for heat losses around the area of the objective.

To standardize the temperature at which *shi*^ts2^ mutants start showing defects in cellularization (Pelissier et al., 2003), the *shi*^ts2^; Spider-GFP combination was imaged at temperatures 28, 30 and 32°C (data not shown). Wild-type embryos showed normal cellularization at these temperatures. Temperature sensitive *shi*^ts2^ mutants show normal early development at 28 and 30°C and a complete disruption of cellularization at 32°C confirming that 32°C is the restrictive temperature for studying defects in *shi*^ts2^ mutants. Multiple wild type (+/+) and mutant *shi*^ts2^ embryos expressing Dyn^WT^-GFP, Dyn^ts2^-GFP and Clc-GFP were imaged at the permissive and restrictive temperature to study the localization of dynamin and clathrin-light chain.

For quantitation, regions of interest were drawn in the syncytial intercap PM in interphase, prophase and metaphase of nuclear cycle 11, 12 or 13 and the fluorescence intensity was extracted in Image J. This intensity was normalized to the cytoplasmic intensity of the fluorescent protein of the respective embryo and then averaged across several embryos to obtain values of intensity for Dyn-GFP or Clc-GFP in intercap regions during the syncytial cycle in wild type and mutant embryos.

Repetitive photobleaching experiments to assess compartmentalization were performed on wild-type and *shi*^ts2^ mutant embryos at restrictive temperatures described by Mavrakis and colleagues (Mavrakis et al., 2008). Briefly a fixed region of interest was drawn on the apical membrane above each nucleus during syncytial cycle 12 and 13 and repetitively photobleached with high intensity Argon laser. Fluorescence depletion was monitored with in the plasma membrane region belonging to same nucleus and in the neighboring plasma membrane (see schematic in Fig. 7M). The average fluorescence intensity in the regions of interest was computed with the help of Image J. For the measurements, the fluorescence intensity of the background was subtracted, the fluorescence values were normalized to the prebleach intensity and the graphs were plotted using Microsoft Excel.

### Generation of α-adaptin mutant germ line clones

The α-adaptin mutant *ada*^5^ in combination with FRT 40A was obtained from Juergen Knoblich, IMP, Vienne, Austria. This was combined with hs-FLP on the X chromosome and then crossed to ovoD; FRT 40A. The progeny were heat pulsed at 38°C for 1 hr during larval, pupal and adult stages. Adults were placed in embryo collection cages and after a 1 hour egg collection were dechorionated and hand devitellinized and stained with DAPI and Phalloidin to visualize actin rings in metaphase and spindles.

## RESULTS

### Spatiotemporal dynamics of dynamin and clathrin during early syncytial mitotic cycles

**Fig. 1.**
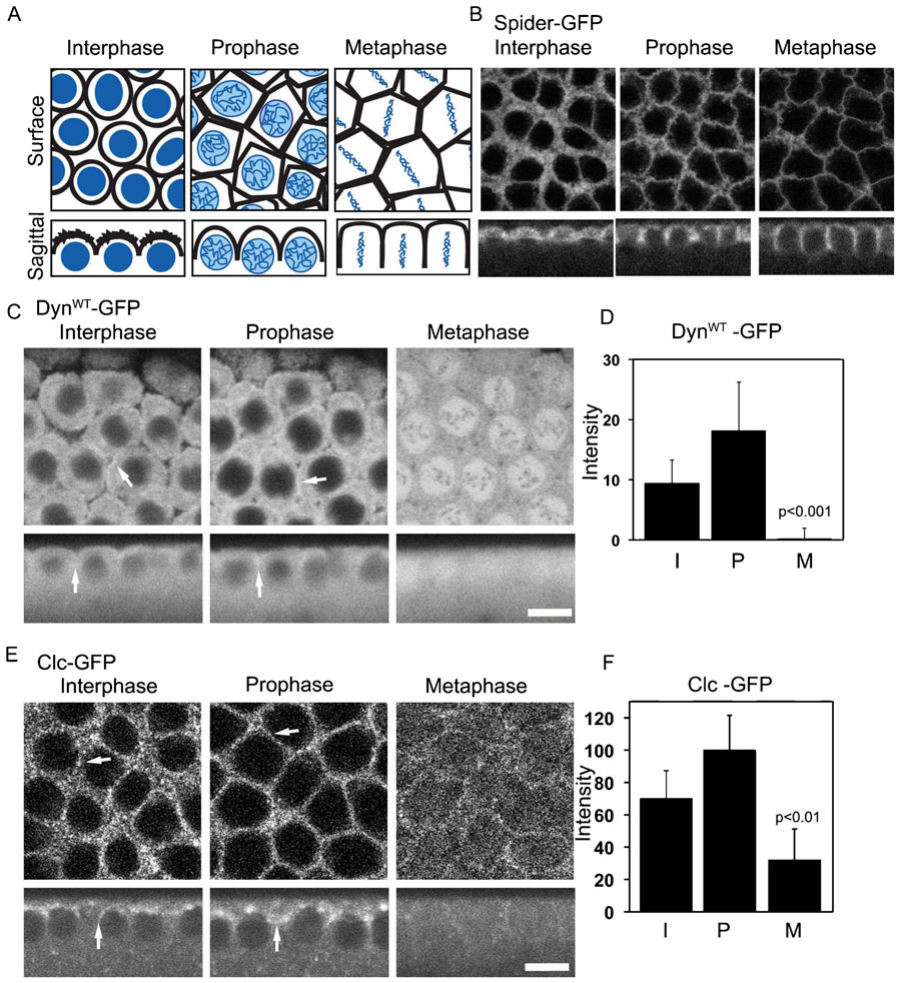
Differential plasma membrane localization of Dynamin and Clathrin in the *Drosophila* syncytial division cycle. (A) Schematic depicting surface and sagittal views of arrangement of nuclei (blue) and plasma membrane (black) in the *Drosophila* syncytial division cycles 11–13. (B) Spider-GFP containing embryos imaged through syncytial cycle 12. Note the change in shape and length of the plasma membrane in surface and sagittal views during the interphase, prophase and metaphase. (C) Nanos-Gal4, Dyn^WT^-GFP was used to express GFP tagged Dynamin in the early syncytial embryo. An increased localization of plasma membrane Dyn^WT^-GFP (marked by white arrows) was seen in interphase and prophase in surface (top panels) and sagittal (bottom panels) views, and was significantly reduced in metaphase. (D) The fluorescence intensity for Dyn^WT^-GFP in the intercap regions relative to the cytoplasm is quantified during the syncytial cycle (n =  40 intercap regions in each stage across 8 embryos). The histogram shows average and error bars are standard deviation. (E) Nanos-Gal4, Clc-GFP was used to express Clc-GFP in the early syncytial embryo. Increased plasma membrane localization of Clc-GFP was seen in interphase and prophase in surface and sagittal views (marked by white arrows) and was significantly reduced in metaphase. (F) The fluorescence intensity for Clc-GFP in the intercap regions relative to the cytoplasm is quantified during the syncytial cycle (n = 20 intercap regions in each stage across 4 embryos). The histogram shows average and error bars show standard deviation. Scale bars = 10 µm.

As a first step toward elucidating dynamin's role in PM furrowing, we performed time-lapse imaging to monitor the distribution of dynamin and clathrin during mitotic cycles 10–13 in the syncytial embryo. The schematic diagram in Fig. 1A and live imaging of embryos containing plasma membrane marker, Spider-GFP in Fig. 1B depicts changes in PM and nuclear organization in surface and sagittal views of the syncytial embryo that are known to occur during cycles of interphase, prophase and metaphase.

Dynamin's distribution was assessed in transgenic flies expressing wild-type (WT) dynamin tagged with green fluorescent protein (GFP) on its C terminus (Dyn^WT^-GFP). These transgenic flies were combined with *nanos*-Gal4 to allow protein deposition of Dyn^WT^-GFP in syncytial embryos. When the transgene is expressed in *shi*^ts^ mutant flies, Dyn^WT^-GFP reverses the temperature sensitive developmental defects characteristic of these flies [such as aberrant cellularization (Pelissier et al., 2003)] (data not shown), indicating that it can functionally replace wild-type dynamin. Further evidence for Dyn^WT^-GFP's suitability as a dynamin probe is that the GFP chimera localizes in neurons in an identical manner to immunostainings for endogenous dynamin in synapses in the larval brain and the neuromuscular junction (data not shown).

In syncytial, wild-type embryos in interphase and prophase, Dyn^WT^-GFP localizes on short furrow membranes in the inter-cap region, as well as in the cytoplasm (Fig. 1C, arrows point to enrichment in intercap region). During metaphase, when intercap membranes invaginate more deeply (see Fig. 1A,B), Dyn^WT^-GFP is depleted from the PM, shifting its distribution to the mitotic spindle zone (Fig. 1C, Metaphase). Measurement of the fluorescent signal along a line through a region that contains the PM (supplementary material Fig. S1B,C) or throughout the intercap zone relative to the cytoplasm across multiple embryos reveals significant reduction of Dyn^WT^-GFP on the PM during metaphase relative to that in interphase and prophase (Fig. 1D). Even though there is a large amount of dynamin in the cytoplasm at all cell cycle stages, the membrane fraction seen in interphase and prophase is significant and specific and is not seen with cytoplasmic RFP (supplementary material Fig. 1F). Cytoplasmic RFP accumulation in the nuclear regions in metaphase is similar to low molecular weight dextrans (Kalpin et al., 1994). Dynamin distribution is also found on the plasma membrane colocalized with amphiphysin in wild type embryos during the syncytial division cycle in prophase though qualitatively there is significant amount of protein in the cytoplasm (supplementary material Fig. S1G). Since Dyn^WT^-GFP effectively reverses the mutant phenotype and mimics the endogenous antibody distribution, we henceforth use the fluorescently tagged transgenes to quantitatively analyze its localization on the plasma membrane in living embryos in different cell cycle phases and at different temperatures.

Wild-type embryos expressing clathrin light chain tagged with GFP (Clc-GFP) were imaged to examine changes in clathrin distribution during the syncytial mitotic cycles. The functionality of the Clc-GFP has been previously demonstrated in the context of Notch endocytosis in the *Drosophila* eye (Hagedorn et al., 2006). Clc-GFP localizes to the intercap PM region during interphase and prophase (Fig. 1E, arrows point to enrichment in intercap zone). This localization is particularly apparent in sagittal views. During metaphase, however, Clc-GFP increases in the cytoplasm or the spindle zone, and is significantly depleted from metaphase furrow plasma membrane. The extent of this change in Clc-GFP quantified in line intensity profiles (supplementary material Fig. 1D,E) and along the intercap PM relative to the cytoplasm from different embryos shows a reduction in Clc-GFP in metaphase furrows (Fig. 1F).

These results suggest that both dynamin and clathrin undergo changes in their PM distribution as embryos progress from interphase to metaphase in the syncytial embryo. The trends, seen in mitotic cycles 10–13, involve a shift of these molecules from being associated with intercap PM in interphase and prophase to being less associated with PM in metaphase. Because endocytic activity requires recruitment of dynamin and clathrin to the PM, the reduction of PM association of these molecules at metaphase suggests that endocytic activity is inhibited at this stage of the nuclear cycle.

### Dynamin-dependent endocytic activity is reduced during metaphase of the syncytial nuclear division cycle

At the restrictive temperature, the *shi*^ts2^ dynamin mutant acutely blocks dynamin-mediated endocytosis, in a reversible manner, by preventing endocytic vesicles from pinching off the PM (Damke et al., 1995; Koenig and Ikeda, 1989; Poodry and Edgar, 1979; van der Bliek and Meyerowitz, 1991; van der Bliek et al., 1993). We used *shi*^ts2^ mutant flies to confirm whether endocytosis is specifically blocked during metaphase of the mitotic cycle in the syncytial embryo. *Shi*^ts2^ mutant embryos were heat shocked at 32°C for 5 min to block endocytosis and accumulate PM endocytic cargo in coated pits. They were then allowed to recover for 2 min at room temperature so that resumption of endocytosis and uptake of PM components could be visualized. The PM components examined in this fashion included DE-cadherin-GFP (Oda and Tsukita, 2001), a known cargo undergoing clathrin-mediated endocytosis (Le et al., 1999) and Spider-GFP, a PM-associated casein kinase (Babu et al., 2002; Frescas et al., 2006).

**Fig. 2.**
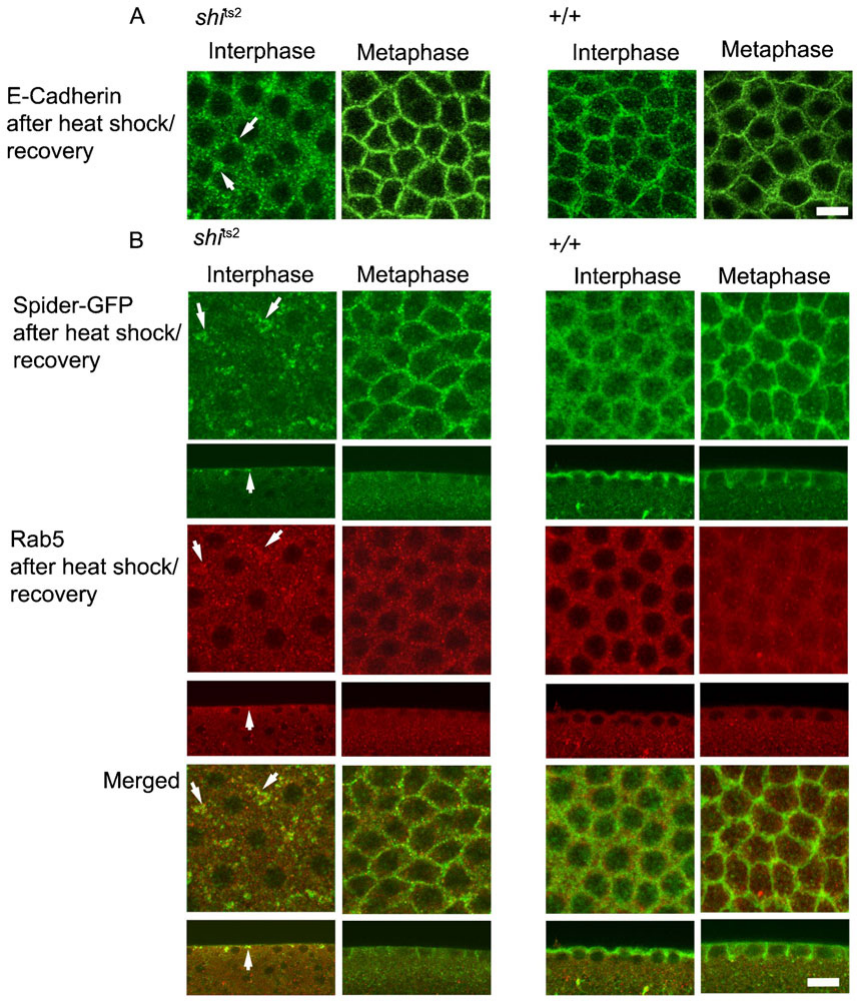
Increased endocytosis of E-cadherin and Spider-GFP in interphase of the syncytial cycle in *Drosophila* embryos. (A) Control and *shi*^ts2^ mutant embryos expressing E-cadherin-GFP were heat pulsed at 32°C for 5 min to accumulate plasma membrane proteins in endocytic pits and released for 2 min at room temperature. Embryos were fixed and stained for GFP. *shi*^ts2^ embryos showed an accumulation of E-cadherin-GFP in vesicular compartments in interphase (left panel) but not in metaphase (right panel). (B) Control and *shi*^ts2^ mutant embryos expressing Spider-GFP were heat pulsed at 32°C to accumulate plasma membrane markers and released for 2 min at room temperature. Embryos were fixed and stained for GFP and Rab5. An increased accumulation of Rab5 and Spider-GFP (marked by white arrows) compartments was seen upon recovery in *shi*^ts2^ mutant embryos in interphase (left panel) but not in metaphase (right panel). Scale bars = 10 µm.

After heat shock in *shi*^ts2^ interphase embryos, DE-cadherin-GFP undergoes significant internalization as compared to control embryos during the 2 min recovery period, appearing in punctate structures in the cytoplasm (Fig. 2A Interphase, *shi*^ts2^). Spider-GFP, which marks the plasma membrane, also redistributes off the PM and onto internal structures during the 2 min recovery period in *shi*^ts2^ embryos (Fig. 2B Interphase, *shi*^ts2^). The internal structures in *shi*^ts2^ embryos containing Spider-GFP localize with immunolabeled Rab5, an early endosome marker. After heat shock in *shi*^ts2^ metaphase embryos, by contrast, virtually no internalization of DE-cadherin-GFP or Spider-GFP occurred during the 2 min recovery (Fig. 2A,B Metaphase, *shi*^ts2^). Instead, the markers remained on the PM. In wild-type embryos, the heat shock/recovery protocol resulted in no change in distribution of any of the markers in interphase or metaphase (Fig. 2A,B, +/+), as expected since the temperature shift from 24°C to 32°C and back again in wild-type embryos does not acutely alter endocytosis.

Together, the results suggest that different PM components undergo dynamin-mediated endocytosis during interphase of the syncytial cycle, whereas endocytosis is decreased during metaphase. This is consistent with dynamin's overall reduction from membranes during metaphase.

### Effects of shi^ts2^ on syncytial cycle localization of dynamin and clathrin

**Fig. 3.**
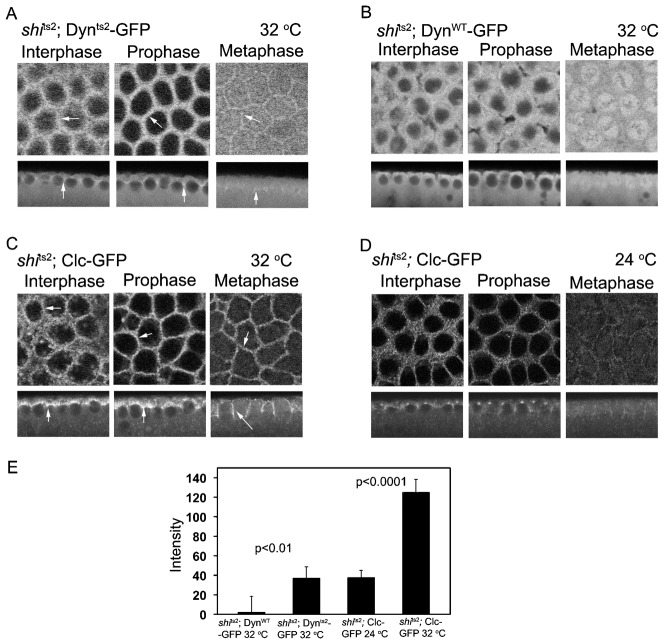
Increased accumulation of Dynamin and Clathrin on the plasma membrane in *shi*^ts2^ mutant embryos at the restrictive temperature. (A) *shi*^ts2^ mutant embryos expressing Dyn^ts2^-GFP were imaged live at the restrictive temperature of 32°C. They showed an increased accumulation of Dyn^ts2^-GFP at the plasma membrane from interphase to metaphase at the restrictive temperature (white arrows). (B) *shi*^ts2^ mutant embryos expressing Dyn^WT^-GFP were imaged live at the restrictive temperature. Dynamin accumulated at the plasma membrane only in interphase and prophase as seen in wild-type embryos expressing Dyn^WT^-GFP in Fig. 1C. (C) *shi*^ts2^ mutant embryos expressing Clc-GFP at restrictive temperatures showed an accumulation of Clc on the membrane from interphase to metaphase of the syncytial cycle (white arrows). (D) *shi*^ts2^ mutant embryos expressing Clc-GFP at the permissive temperatures showed an accumulation of Clc-GFP on the membrane only in interphase and prophase as seen in wild-type control embryos in Fig. 1E. (E) The fluorescence intensity with respect to the cytoplasm in the intercap region in metaphase of the syncytial cycle was computed for the respective embryos. (Dyn-GFP: n = 40 metaphase furrows across embryos for B and 15 metaphase furrows across 3 embryos for A; Clc-GFP n = 15 metaphase furrows across 3 embryos for D and 20 furrows across 4 embryos for C). Histogram depicts average and error bars represent standard deviation. Scale bars = 10 µm.

We next examined dynamin's distribution in embryos expressing the *shi*^ts2^ mutation. A GFP-tagged dynamin transgene containing a point mutation in *shi*^ts2^ (G141S) (van der Bliek and Meyerowitz, 1991), called Dyn^ts2^-GFP, was expressed in *shi*^ts2^ flies. At the restrictive temperature of 32°C, embryos in interphase, prophase or metaphase all show Dyn^ts2^-GFP localizing to the PM in the inter-cap region, in addition to its cytoplasmic pool (Fig. 3A, arrows point to labeling in inter-cap zone). In the Dyn^ts2^-GFP, *shi*^ts2^ flies there was also reduced redistribution of dynamin into the mitotic spindle region, as found for Dyn^WT^-GFP expressed in *shi*^ts2^ flies at 32°C (Fig. 3B) or in wild-type flies at 24°C (see Fig. 1C). The intensity of dynamin remaining at the metaphase furrow region relative to the cytoplasm was quantified across multiple embryos at the restrictive temperature in control and mutant flies and found to be significantly higher in mutant flies at the restrictive temperature (Fig. 3E).

Clathrin behavior in the *shi*^ts2^ mutant flies was next examined using Clc-GFP expressed in *shi*^ts2^ flies. At the restrictive temperature, Clc-GFP was enriched at the intercap PM during all mitotic phases (Fig. 3C). This contrasts with Clc-GFP's behavior at the permissive temperature. There, Clc-GFP largely dissociates from the intercap region (Fig. 3D), resembling Clc-GFP in wild-type embryos in metaphase (see Fig. 1E). This change was quantified across the metaphase furrow relative to the cytoplasm in multiple *shi*^ts2^ embryos expressing Clc-GFP imaged at the permissive and restrictive temperature. A significant increase in Clc-GFP was found to be remaining at the metaphase furrow in *shi*^ts2^ embryos at the restrictive temperature (Fig. 3E).

Together, the results suggest that when dynamin activity is blocked in *shi*^ts^ embryos at the restrictive temperature, both Dyn^ts2^-GFP and Clc-GFP keep their association with furrows throughout the mitotic cycle. This is similar to previous observations in epithelial cells expressing the dynamin mutations in the GTPase domain (Chua et al., 2009) and also during application of the dynamin inhibitor, dynasore, to mammalian cells (Macia et al., 2006). There, dynamin remains tightly associated with the PM in clathrin-coated pits. The pits cannot pinch off the PM and are possibly enriched in endocytic cargo.

### Morphogenetic defects in metaphase furrow formation in the syncytial cycle in dynamin mutants

**Fig. 4.**
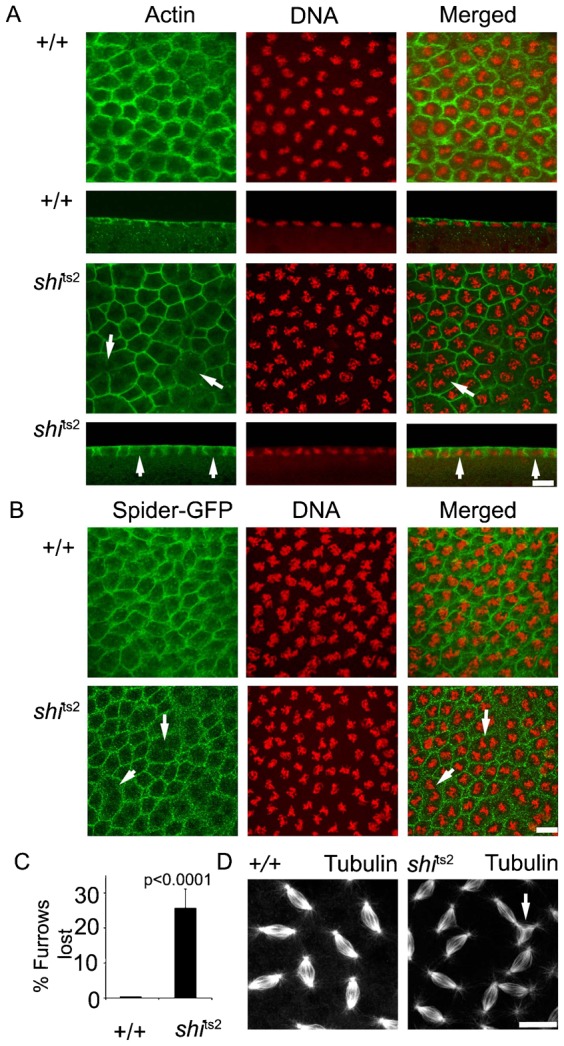
*shi*^ts2^ mutant embryos show a loss of metaphase furrows and tripolar metaphase spindles. *shi*^ts2^ mutant embryos were heat pulsed at 32°C, fixed and stained for actin with phalloidin (green) and DNA (red) (A), plasma membrane (Spider-GFP, green) (B) and metaphase spindles (tubulin) (D). White arrows in A and B point to regions with disrupted metaphase furrows and in D to tripolar spindles in *shi*^ts2^ mutant embryos at the restrictive temperature (D). Quantitation of the phenotype shows that each embryo looses 25% of the metaphase furrows. n = 10 for wild-type, n = 13 for *shi*^ts2^ mutant embryos, error bars indicate standard deviation (C). Scale bars = 10 µm.

We next looked for morphogenetic consequences of having dynamin fail to redistribute off the PM in temperature-restricted *shi*^ts2^ embryos during the syncytial division cycle. Among the key morphological events that occur during metaphase is redistribution of actin to the intercap zone in prophase for driving metaphase furrow formation around dividing nuclei (Miller et al., 1985). These actin rings are seen in association with ingressed metaphase furrows in wild-type embryos that are fixed and immunostained for actin and DNA (Fig. 4A, top panels). When *shi*^ts2^ embryos are fixed at the restrictive temperature, however, metaphase actin rings are often incompletely formed (Fig. 4A, bottom panels, see arrows). Moreover, some rings surround more than one condensed mass of DNA (Fig. 4A, bottom panels, see arrows). The appearance of incomplete actin rings, and rings around more than one DNA mass, indicates that actin redistribution during metaphase furrow ingression is defective in *shi*^ts2^ mutants.

To assess if the PM at the metaphase furrow is similarly disrupted in the mutant embryo, we used Spider-GFP as a PM marker together with DNA labeling to examine furrows. Approximately 25% of furrows in *shi*^ts2^ embryos are lost at the restrictive temperature in patches across each embryo (Fig. 4C). Moreover, those metaphase furrows present surround many DNA elements, unlike in control embryos (Fig. 4B, see arrows). Double labeling of actin and Spider showed complete overlap between these markers, with regions of embryos lacking actin rings also lacking PM furrows labeled by Spider-GFP (supplementary material Fig. S2A,B). Thus, actin ring formation and ingression of PM furrows during metaphase are jointly disrupted in temperature-restricted *shi*^ts2^ embryos. Similar defects in metaphase furrow formation are also seen in germline clones of α-adaptin mutants *ada*^5^ (supplementary material Fig. S2C) which encodes a protein essential for dynamin mediated endocytosis at the PM.

Maintenance of bipolar spindles during metaphase potentially depends on PM furrowing (Afshar et al., 2000; Stevenson et al., 2002), so we examined the occurrence of abnormal mitotic spindles in temperature-restricted *shi*^ts2^ embryos. Incidences of tripolar spindles are seen in temperature-restricted *shi*^ts2^ embryos relative to wild-type embryos (Fig. 4D, see arrows). Altogether, the results indicated that preventing dynamin from redistributing off the PM in temperature-restricted *shi*^ts2^ embryos interferes with ingression of metaphase furrows.

### Actin assembly into contractile rings at ingressing furrows rather than cap disassembly is affected in *shi^ts^*^2^ embryos

Previous work in early *Drosophila* embryos has shown that when actin-based machinery (such as Diaphanous and Arp2/3) is disrupted, leading to defects in furrow ingression, actin caps have not disassembled at prophase (Afshar et al., 2000; Stevenson et al., 2002). To test whether actin caps likewise fail to disassemble in *shi*^ts2^ embryos, we imaged actin dynamics in live embryos progressing from interphase through metaphase after a shift to the restrictive temperature. Actin was monitored in these embryos by following the distribution of GFP-tagged moesin, an Ezrin-radixin-moesin (ERM) family member with a conserved actin-binding domain important for orchestrating actin redistribution to rings in the embryo syncytial cycle (Kiehart et al., 2000).

**Fig. 5.**
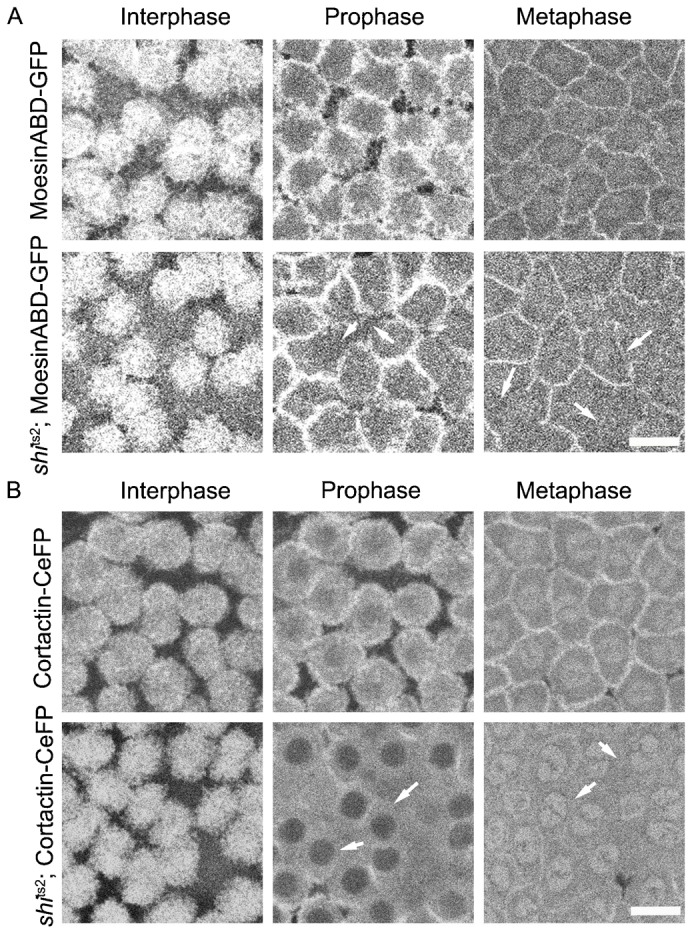
Actin regulatory proteins Moesin and Cortactin are decreased on the metaphase furrow in *shi*^ts2^ mutant embryos at the restrictive temperature. (A) MoesinABD-GFP (Moesin actin binding domain-GFP) was used to mark actin in control and *shi*^ts2^ mutant embryos during the syncytial cycle. MoesinABD-GFP labels actin caps in interphase. In both control and *shi*^ts2^ mutant embryos there is a redistribution of actin to the lateral membrane in prophase and metaphase. *shi*^ts2^ mutant embryos show an incomplete redistribution of actin to the lateral membrane in both prophase and metaphase (white arrows). (B) Cortactin-CeFP, a Dynamin binding protein, was expressed in control and *shi*^ts2^ mutant embryos using nanos-Gal4. Cortactin marked the actin cap in interphase, relocated to the lateral membrane in prophase and to metaphase furrow membranes in metaphase. In *shi*^ts2^ mutant embryos at the restrictive temperature, Cortactin failed to relocate to the lateral membrane in prophase and metaphase (white arrows). Scale bars = 10 µm.

Confocal imaging of Moesin-GFP in wild-type embryos reveals labeling of actin caps in interphase and actin rings in metaphase (Fig. 5A, top row). In *shi*^ts2^ embryos expressing Moesin-GFP shifted to the restrictive temperature, actin caps disassemble in prophase normally. However, aberrant ring structures are seen in prophase and metaphase (Fig. 5A, bottom row, arrows point to areas without actin rings). This indicated that perturbation of dynamin dynamics in *shi*^ts2^ embryos interferes with actin assembly into rings at furrows, rather than with disassembly of actin caps.

### Dynamin release from metaphase furrows correlates with Cortactin association with furrows

Cortactin is an F-actin binding protein involved in actin remodeling at the PM and is known to associate with dynamin (Daly, 2004; McNiven et al., 2000). To understand why disrupting dynamin dynamics in temperature-restricted *shi*^ts2^ embryos interferes with actin contractile ring assembly and ingression of metaphase furrows, we imaged wild-type and *shi*^ts2^ transgenic lines expressing a chimera of cortactin tagged with Cerulean fluorescent protein (Cortactin-CeFP).

In wild-type syncytial embryos in interphase, Cortactin-CeFP associates with actin caps like moesin (Fig. 5A). It then redistributes to the intercap region in prophase (Fig. 5B). In metaphase, rather than dissociating from ingressed furrows as seen for dynamin (see Fig. 1C), cortactin remains associated with the furrow PM (Fig. 5B). In *shi*^ts2^ mutant embryos at the restrictive temperature, Cortactin-CeFP association with the intercap region in prophase is reduced (Fig. 5B, prophase, arrows point to areas without intercap labeling) and largely dissociates from membranes during metaphase, redistributing into the mitotic spindle zone (Fig. 5B, metaphase, arrows point to spindle enrichment). The effect is thus opposite to that seen for dynamin, which enriches on furrow membranes in temperature-restricted *shi*^ts2^ mutant embryos in both prophase and metaphase (see Fig. 3A).

These results suggest that actin contractile ring assembly during prophase and furrow ingression are disrupted in temperature-restricted *shi*^ts2^ mutant embryos because of actin-binding proteins, like moesin and cortactin, failing to associate with furrow membranes. This could be because the *shi*^ts2^ mutation leads to membrane proteins required for actin remodeling being sequestered in non-severed coated vesicles. This interpretation predicts that other actin-regulatory molecules controlling furrow dynamics also may be mislocalized in temperature-restricted *shi*^ts2^ embryos.

### Mislocalization of syncytial actin regulatory machinery in temperature-restricted *shi^ts2^* embryos

**Fig. 6.**
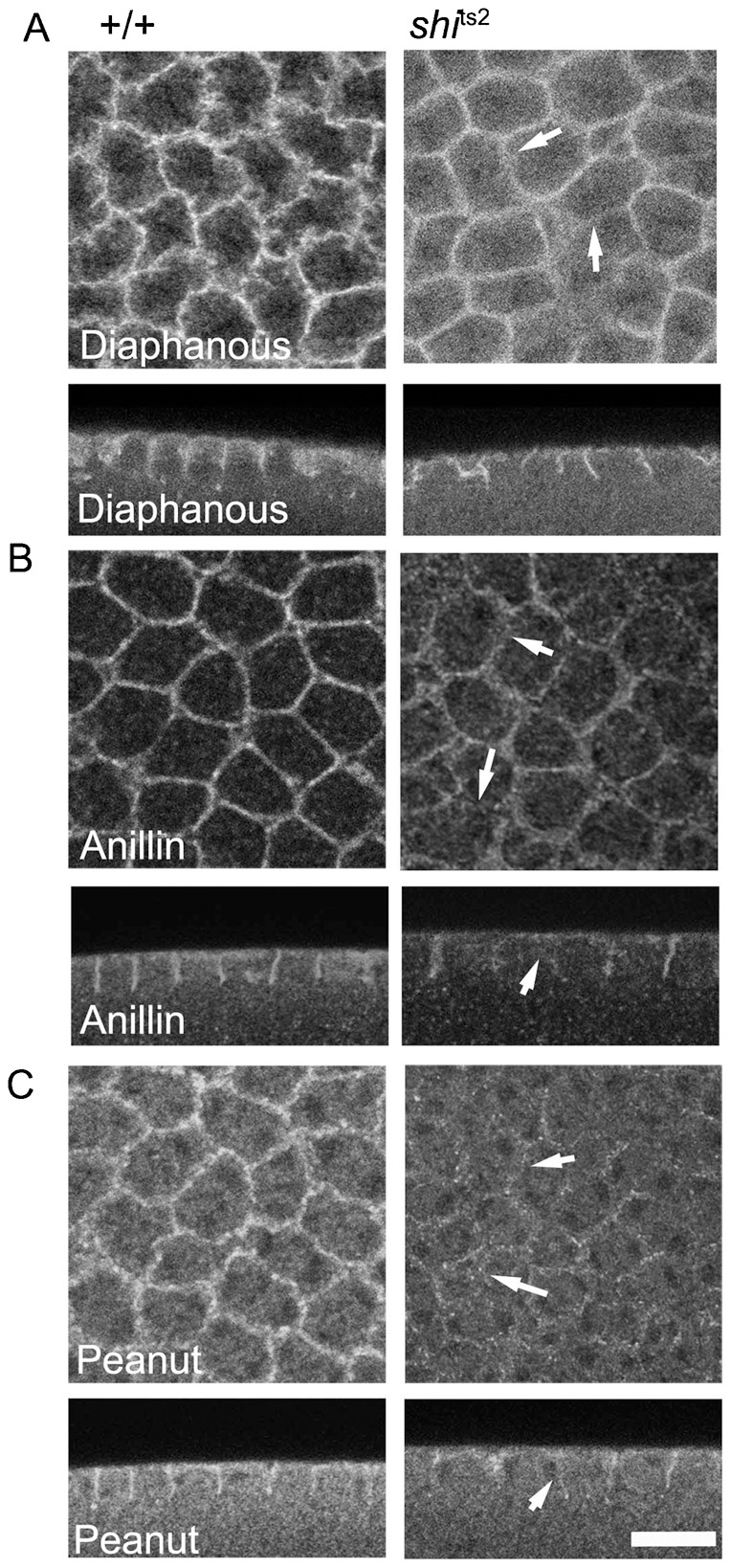
Diaphanous, Anillin and Peanut are reduced on the metaphase furrow membrane in *shi*^ts2^ mutant embryos at restrictive temperatures. Diaphanous, Anillin and Peanut immunostainings were done in control and *shi*^ts2^ embryos after fixation at the restrictive temperature. There was a decrease in immunostaining of Diaphanous (A), Anillin (B) and Peanut (C) on the metaphase furrow membrane. Scale bars = 10 µm.

We tested this prediction by examining in *shi*^ts2^ embryos the distribution of several actin-regulatory molecules essential for metaphase furrow formation. Diaphanous is a formin homology domain containing protein necessary for furrow ingression in metaphase, potentially important for bundling actin during actomyosin-driven contraction of metaphase furrows (Afshar et al., 2000). Antibody labeling for Diaphanous shows localization on ingressed metaphase furrows in wild-type embryos but decreased labeling in *shi*^ts2^ mutant embryos at the non permissive temperature (Fig. 6A, arrows in *shi*^ts2^ image point to areas with significantly reduced labeling).

Diaphanous mutants show a reduction of the actin regulatory proteins, Anillin and Peanut on the furrow membranes (Afshar et al., 2000). The PH domain-containing protein, Anillin, was next examined. It binds and bundles actin filaments (Field and Alberts, 1995) and also recruits septins to actin bundles (Kinoshita et al., 2002) and membranes (Field et al., 2005). Antibody labeling for Anillin in wild-type syncytial embryos reveals enrichment in ingressed metaphase furrows (Fig. 6B, +/+), as previously shown (Field and Alberts, 1995; Silverman-Gavrila et al., 2008). Less enrichment is seen in *shi*^ts2^ embryos at nonpermissive temperature (Fig. 6B, *shi*^ts2^, arrows point to areas of reduced labeling).

The septin protein, Peanut, was also examined. Septins are self-assemblying GTPases, essential for the stabilization of the metaphase and the cellularization furrows in the syncytial *Drosophila* embryo (Adam et al., 2000; Silverman-Gavrila et al., 2008). Antibody labeling reveals that Peanut localizes to deeply ingressed metaphase furrows in wild-type embryos (Fig. 6C, +/+ Peanut). This localization is reduced in *shi*^ts2^ embryos at the restrictive temperature (Fig. 6C, *shi*^ts2^, arrows point to areas of reduced labeling).

Together, these results suggest that proper recruitment/stabilization of Diaphanous, Anillin and Peanut at the metaphase furrow, like cortactin, is dependent on changes in dynamin distribution. That is, dynamin release from membranes in metaphase correlates with enrichment of actin regulatory molecules and stabilization of furrow membranes to orchestrate furrow ingression. In temperature-restricted *shi*^ts2^ embryos, PM proteins are sequestered in non-severed coated vesicles, unable to bind the actin regulatory molecules, so furrow ingression does not occur. Given that dynamin redistribution is important for metaphase furrow ingression, we next turned to address in what way dynamin is important during interphase of the syncytial mitotic cycle.

### *shi^ts2^* embryos show defects in polarity and loss of PM compartmentalization during interphase of the syncytial cell cycle

**Fig. 7.**
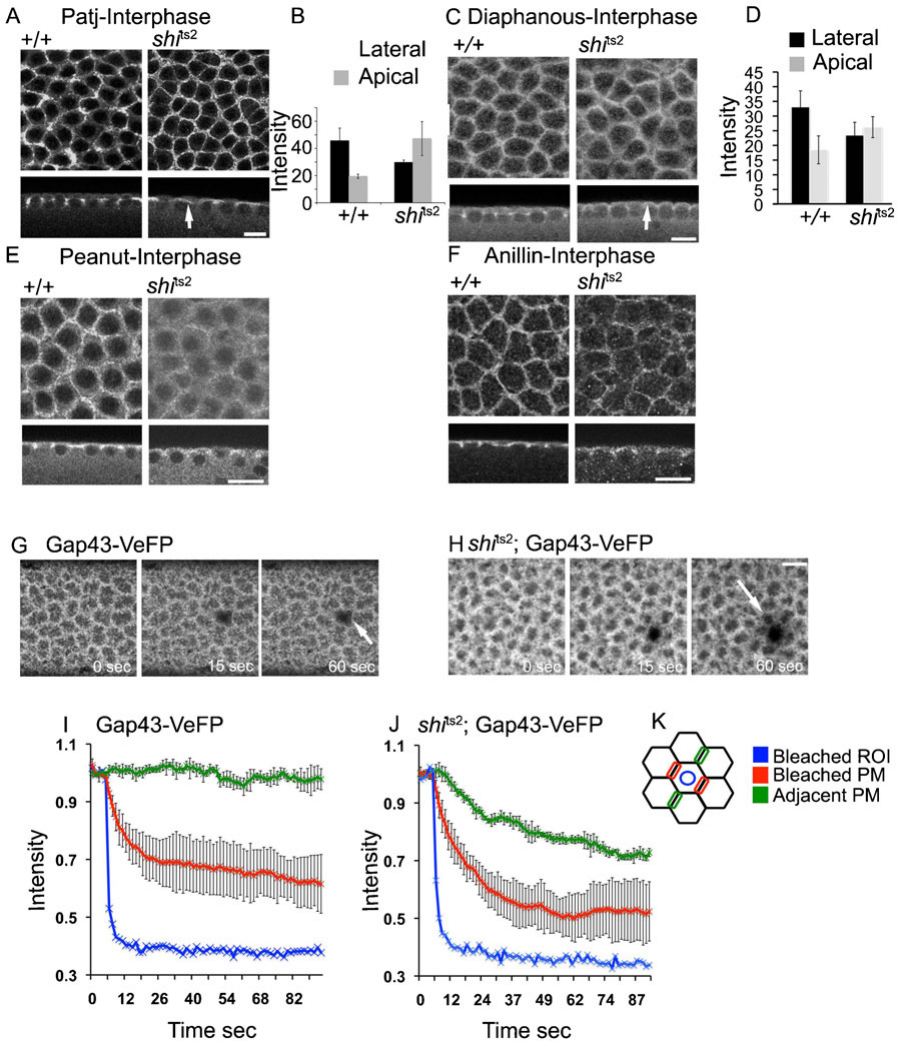
Dynamin mutants show a defect in the polarized distribution of proteins in interphase and in the compartmentalization of the interphase plasma membrane. (A–D) Control wild-type and *shi*^ts2^ mutant embryos were fixed at the restrictive temperature and immunostained for Diaphanous and Patj. There was a decrease in Patj and Diaphanous in lateral regions shown in sagittal sections compared to the control embryos (A and C). Intensity of Diaphanous and Patj in apical and lateral regions from sagittal sections are quantified in B and D. Histogram represents average and error bars show standard deviation. (E,F) Control wild-type and *shi*^ts2^ mutant embryos were fixed at the restrictive temperatures and immunostained for Anillin and Peanut. A decrease in immunostaining was seen in both surface and sagittal sections. (G–K) Control and *shi*^ts2^ embryos in interphase of the syncytial division expressing GAP43-VeFP were assayed for compartmentalization at the restrictive temperature. A circular region containing the apical membrane above each interphase nucleus (K, blue circle) was repetitively photobleached, and fluorescence was measured over time on the lateral membranes surrounding either the same nucleus (K, red) or the adjacent nucleus (K, green). Repetitive photobleaching showed that the membrane compartmentalization of GAP43-VeFP was lost in *shi*^ts2^ mutant embryos compared to control embryos (G,H and quantitation in I,J). Each point is an average from a representative bleaching experiments and error bars indicate standard deviation in this FLIP, n = 3. Scale bars = 10 µm.

Dynamin dynamically associates with furrow membranes during interphase, catalyzing scission of endocytic vesicles carrying membrane components. We next assessed the polarized distribution of PM proteins during interphase of the syncytial cycle in *shi*^ts2^ mutants. Antibody labeling for Patj [a junctional membrane protein (Bhat et al., 1999)], Diaphanous, Anillin and Peanut, revealed a significant steady-state enrichment of these proteins in lateral membranes in wild-type embryos in interphase (Fig. 7A–F). In *shi*^ts2^ embryos at the restrictive temperature, however, Patj and Diaphanous lost their polarized distribution. This could be seen in sagittal views (Fig. 7A,C) and by quantitation of apical and lateral labeling intensities (Fig. 7B,D). Anillin and Peanut were reduced on the interphase PM (Fig. 7E,F).

Compartmentalization of the syncytial embryo PM into distinct units above individual nuclei is likely to be dependent on the polarized distribution of junctional and actin cytoskeleton regulatory proteins on the PM (Mavrakis et al., 2009). Because such polarized distribution of these proteins is lost in *shi*^ts2^ embryos at the restrictive temperature, we tested whether this leads to loss of compartmentalization. A lipid anchored PM marker, Gap43-VeFP (Venus fluorescent protein) (Mavrakis et al., 2009), was expressed in wild-type or temperature-restricted *shi*^ts2^ syncytial embryos. A circular region belonging to the apical membrane above each nucleus was then repetitively photobleached (blue circle) (Schematic, Fig. 7K) to assess the ability of the protein to diffuse across the PM of the embryo. Photobleaching was monitored on the lateral membrane surrounding either the same nucleus (red regions) or on the membrane surrounding the adjacent nucleus (green regions).

In wild-type embryos, fluorescence depletes rapidly from the lateral membrane surrounding the nucleus near the site of photobleaching and more slowly from the PM surrounding neighboring nuclei, indicative of restricted diffusion of Gap43-VeFP to areas within the junctional belt region (Fig. 7G and quantitation in Fig. 7I). In *shi*^ts2^ embryos at the restrictive temperature, by contrast, fluorescence depletes rapidly from both the lateral membrane surrounding the nucleus adjacent to the site of photobleaching and the PM surrounding neighboring nuclei (Fig. 7H and quantitation in Fig. 7J). The diffusion barrier across the junctional belt is therefore lost in *shi*^ts2^ embryos at the restrictive temperature.

These results indicate that impaired scission of vesicles in temperature-restricted *shi*^ts2^ embryos in interphase both disrupts PM polarity and leads to loss of PM compartmentalization across the embryo. Developmental control of dynamin localization and dynamics, therefore, is essential for embryo morphogenesis during both metaphase and interphase of the syncytial mitotic cycle.

## DISCUSSION

The establishment of PM furrows as discrete compartments surrounding individual nuclei and their mitotic ingression/regression dynamics is an essential mechanism for *Drosophila* embryo compartmentalization before cellularization (Mavrakis et al., 2009). It allows each nucleus in the embryo to function almost like an independent cell unit and is indispensable for keeping mitotic spindles isolated across the syncytial embryo. While much work has led to our understanding of the role of cytoskeletal regulators and membrane traffic components in setting up furrow organization (Afshar et al., 2000; Cao et al., 2008; Karr and Alberts, 1986; Kellogg et al., 1988; Riggs et al., 2003), the specific molecules that are differentially associated with the interphase and metaphase furrow have not been identified. Here, we provide evidence that dynamin GTPase is one such molecule important for furrow ingression. We discuss the endocytic function of dynamin during the syncytial cycle as 1) a potential mediator of morphogenetic change leading to metaphase furrow formation, 2) a possible regulator of adhesive protein build up in metaphase furrow formation and 3) functionally important for compartmentalization in the syncytial *Drosophila* embryo.

### Dynamin endocytosis is controlled during the *Drosophila* syncytial division cycle

We found that dynamin was localized on short PM furrows during interphase, but redistributed off furrows and into the mitotic spindle region in metaphase. While it is unknown how syncytial embryos mediate this switch in dynamin localization between interphase and metaphase, its morphogenetic consequences are significant. Temperature-restricted *shi*^ts2^ embryos blocked the dynamin function and caused dynamin to remain PM-bound. We found that furrow organization and ingression in metaphase was disrupted, and that the embryo PM in interphase lost its polarity and compartmentalized character. As discussed below, we propose that these dramatic phenotypes arise because of the central role that endocytic control plays in coordinating actomyosin machinery for the developmental regulation of furrowing and PM organization in the early *Drosophila* embryo.

The dynamic association of dynamin with furrow membranes during interphase enables endocytic vesicles to be severed from the PM. This process drives the endosomal circulatory system that the embryo uses to fine-tune the amount and type of membrane proteins on its furrows. Among the proteins undergoing such circulation is E-cadherin. In an endocytic assay in the interphase syncytial embryo, we found that E-cadherin undergoes internalization after release from an endocytic block. Given E-cadherin's ability to cause membranes to adhere to each other through homotypic clustering, and its interaction with actin-regulatory molecules (Cox et al., 1996; Le et al., 1999; Tepass et al., 1996), constitutive uptake of E-cadherin could help explain how furrows are short in interphase. That said, maintaining steady-state levels of E-cadherin, as well as other actin regulatory molecules, on interphase furrows appears to be essential for furrows to function in PM polarity and compartmentalization in the embryo. This was revealed in our experiments using temperature-restricted *shi*^ts2^ embryos, where proteins become trapped in coated vesicles unable to sever from the PM.

That the embryo uses dynamin as a switch to control endocytosis was suggested by the fact that during metaphase, endocytosis is blocked and dynamin function is inhibited at the PM. Other key endocytic regulators, like clathrin, share this characteristic, suggesting a potential overall change in PM lipid composition at mitosis might drive such redistribution. Along these lines, dynamin recruits to the PM through a PH domain that binds specific phospholipids in the PM (Achiriloaie et al., 1999). Spindle proteins having affinity for dynamin and clathrin (Royle et al., 2005; Thompson et al., 2002), may also underlie dynamin's and clathrin's redistribution to the spindle matrix during metaphase.

The consequence of having dynamin and other endocytic regulators redistributing off the PM in metaphase embryos is that recycling membrane proteins, like E-cadherin, build up on the furrow PM. This, in turn, leads to buildup of cytosolic components that either cross-link membranes to stabilize furrow canals, and/or couple membrane proteins to the actomyosin contractile system to drive furrow invagination. This mechanism for furrow stabilization/ingression during metaphase by restraint of endocytosis is similar to what embryos use to elongate furrows during cellularization (Sokac and Wieschaus, 2008). There, the controlled expression of the zygotic gene *nullo*, an actin binding protein that regulates vesicle scission to restrain endocytosis, plays a primary role. Endocytic modulation, potentially through dynamin, is also likely involved in the developmental regulation of dorsoventral and anteroposterior morphogen gradients in the *Drosophila* embryo. This is because receptor downregulation of the Toll (Huang et al., 2010) and Torso (Lloyd et al., 2002) receptors by endocytosis is required for attenuating their signal transduction.

### Dynamin is a candidate for regulation of actin mediated morphogenetic changes in metaphase furrow in the *Drosophila* syncytium

Preventing endocytic modulation during metaphase in temperature-restricted *shi*^ts2^ embryos in our experiments led to Cortactin, Diaphanous, Anillin and Peanut either mislocalizing or dissociating from the furrow PM. This loss of actin remodeling proteins has been previously observed in other membrane trafficking mutant embryos such as *rab11* (Riggs et al., 2003). Also, the specific loss of Anillin and Peanut is similar to that seen in Diaphanous mutant (*dia*^5^) embryos (Afshar et al., 2000). In all cases, the furrow PM does not ingress properly, leading to a loss of metaphase furrows between adjacent spindles. We propose that these effects emerge in the temperature-restricted *shi*^ts2^ embryos, because dynamin remains PM-associated, unable to bind or hydrolyze its GTP. This directly or indirectly prevents vesicle-associated PM proteins, coated with clathrin and other endocytic effectors (Chua et al., 2009; Macia et al., 2006), from cross-linking with F-actin binding proteins and actomyosin machinery. Consequently, furrow stabilization/ingression is disrupted.

This interpretation fits well with prior data showing endocytosis is critical for maintaining polarity in various contexts such as junctional polarity in epithelial cells, distribution of polarity proteins in the budding yeast and developing C. elegans embryo (Marco et al., 2007; Shivas et al., 2010). Endocytosis and recycling of polarity proteins is essential for maintaining a dynamic distribution of polarity proteins at the plasma membrane (Marco et al., 2007). That dynamin, in particular, plays a critical role in this process was shown in studies where dynamin removal led to a loss of polarity formation in MDCK cells (Chua et al., 2009) and to Par proteins mislocalizing in *C. elegans* embryos (Nakayama et al., 2009). Interestingly, when an inactive form of dynamin (i.e., Dyn2K44A) is overexpressed in epithelial cells, the cells undergo apical constriction (Chua et al., 2009), in which the actomyosin belt surrounding the junctional region contracts. In the wild type syncytial embryo, however, loss of dynamin recruitment leads to progression of the metaphase furrow. Endocytic control thus appears to be intimately and differentially linked to coordinating membrane and actin filament remodeling during both mammalian epithelial cells and early *Drosophila* morphogenesis.

### Dynamin function is needed for syncytial compartmentalization

Finally, our findings are relevant to control of PM compartmentalization in the syncytial *Drosophila* embryo. Key junctional and actin remodeling factors, including Patj, Diaphanous, Peanut and Anillin, all lost their polarized PM distribution in interphase of the syncytial *Drosophila* embryo. The distribution of junctional and actin remodeling factors in the interphase furrow, therefore, is dependent on the activity of endocytic regulators, like dynamin. The polarized distribution of actin regulatory proteins and junctional proteins, including septins that help form diffusion barriers (Caudron and Barral, 2009), is known to be crucial for forming a barrier between adjacent nuclei in the syncytial embryo. Indeed, disruption of this barrier by global disruption of actin using actin-depolymerizing drugs results in the spread of the Dorsal morphogen gradient (Mavrakis et al., 2009). We found that disrupting dynamin dynamics in the temperature-restricted *shi*^ts2^ embryo leads to randomization/depletion of key actin regulatory proteins at furrows and loss of PM compartmentalization. Hence, the embryo's tight control of endocytosis, through modulation of dynamin localization, underlies PM compartmentalization in the *Drosophila* syncytium. A future analysis of the specific control of actin remodeling proteins on compartmentalization will help understand their function in syncytial organization.

## Supplementary Material

Supplementary Material
