# Supplementary material for: Dynamin regulates metaphase furrow formation and plasma membrane compartmentalization in the syncytial Drosophila embryo
Source: Biol Open. 2015 Feb 6;4(3):301–11. doi: 10.1242/bio.20149936 (PMC4359736; doi:10.1242/bio.20149936)
Supplement: Supplementary Material [file supp_4_3_301__index.html]

Dynamin regulates metaphase furrow formation and plasma membrane compartmentalization in the syncytial Drosophila embryo — Dynamin regulates metaphase furrow formation and plasma membrane compartmentalization in the syncytial Drosophila embryo — Supplementary Material 

# Dynamin regulates metaphase furrow formation and plasma membrane compartmentalization in the syncytial *Drosophila* embryo

## bio.20149936 Supplementary Material

**Files in this Data Supplement:**

- Supplementary Material - Richa Rikhy et al. doi: 10.1242/bio.20149936
