# Supplementary material for: Dynamin regulates metaphase furrow formation and plasma membrane compartmentalization in the syncytial Drosophila embryo
Source: Biol Open. 2015 Feb 6;4(3):301–11. doi: 10.1242/bio.20149936 (PMC4359736; doi:10.1242/bio.20149936)
Supplement: Supplementary Material [file supp_bio.20149936_bio.20149936-s1.pdf]

Biology Open (2015) 000, 1–11 doi:10.1242/bio.20149936

Supplementary Material

Richa Rikhy et al. doi: 10.1242/bio.20149936

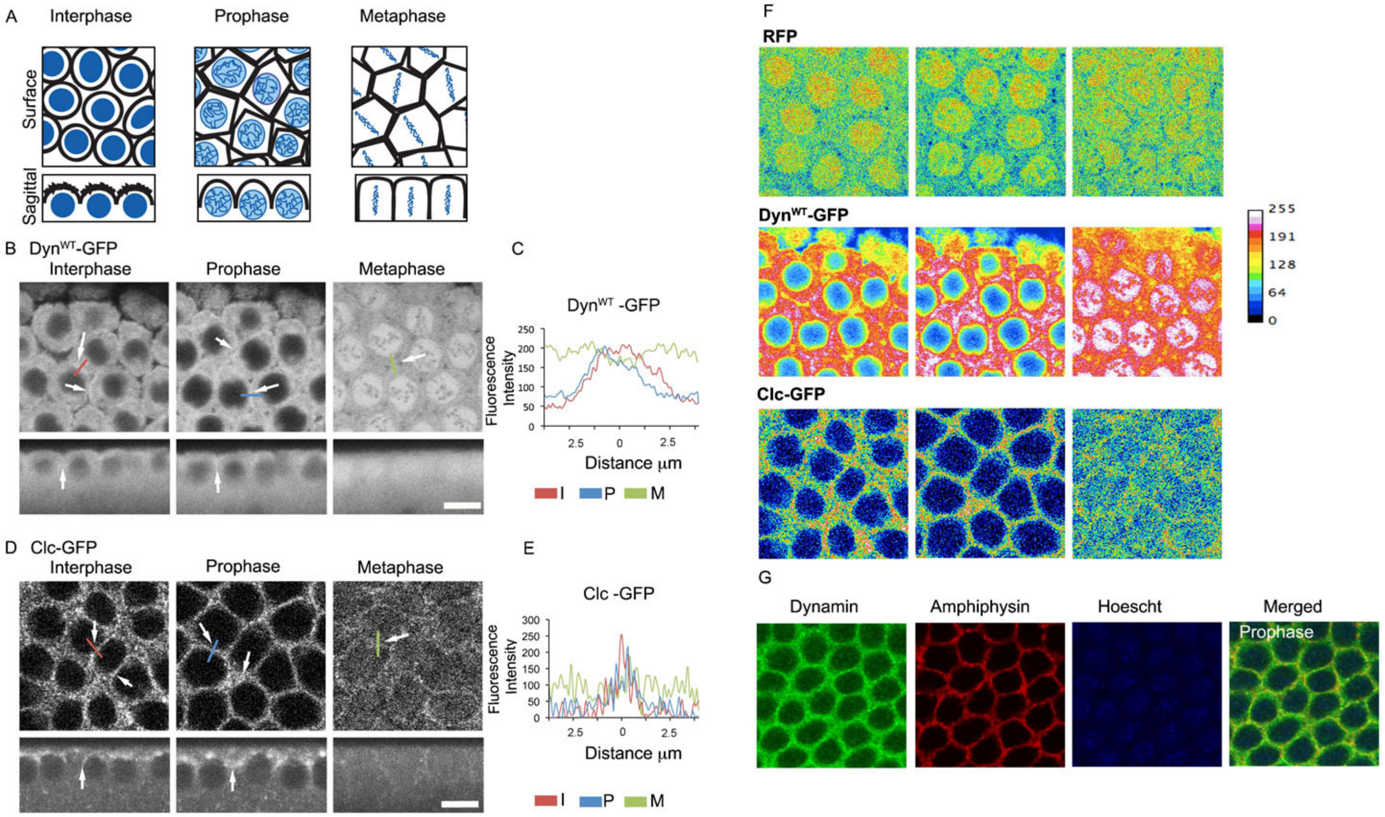

Fig. S1. Differential plasma membrane localization of Dynamin and Clathrin in the *Drosophila* syncytial division cycle. (A) Schematic depicting surface and sagittal views of arrangement of nuclei (blue) and plasma membrane (black) in the *Drosophila* syncytial division cycles 11–13. (B) Nanos-Gal4, Dyn^{WT}-GFP was used to express GFP tagged Dynamin in the early syncytial embryo. An increased localization of plasma membrane Dyn^{WT}-GFP (marked by white arrows) was seen in interphase and prophase in surface (top panels) and sagittal (bottom panels) views, and was significantly reduced in metaphase. (C) The fluorescence intensity profile across a line (B) through the intercap membrane region is plotted for interphase, prophase and metaphase embryos. (D) Nanos-Gal4, Clc-GFP was used to express Clc-GFP in the early syncytial embryo. Increased plasma membrane localization of Clc-GFP was seen in interphase and prophase in surface and sagittal views (marked by white arrows) and was significantly reduced in metaphase. (E) The fluorescence intensity profile across a line (D) through the intercap membrane region is plotted for interphase, prophase and metaphase embryos. (F) Distribution of cytoplasmic RFP is compared to Clc-GFP and Dyn^{WT}-GFP using a multicolored look up table. Note that there is an increased signal for Dyn^{WT}-GFP and Clc-GFP in intercap regions relative to the cytoplasm whereas for cytoplasmic RFP the increased signal appears in the nucleus. (G) Wild type embryos in prophase of syncytial cycle 13 were immunostained Dynamin (green), Amphyphysin (red) and Hoescht (blue). Note the accumulation of Dynamin in the plasma membrane regions where amphiphysin is present in prophase. A quantitative documentation of accumulation in different stages of the syncytial cycle is available from the live imaging in Fig. 1D and supplementary material Fig. S1C. Scale bars=10 µm.

Biology Open (2015) 000, 1–11 doi:10.1242/bio.20149936

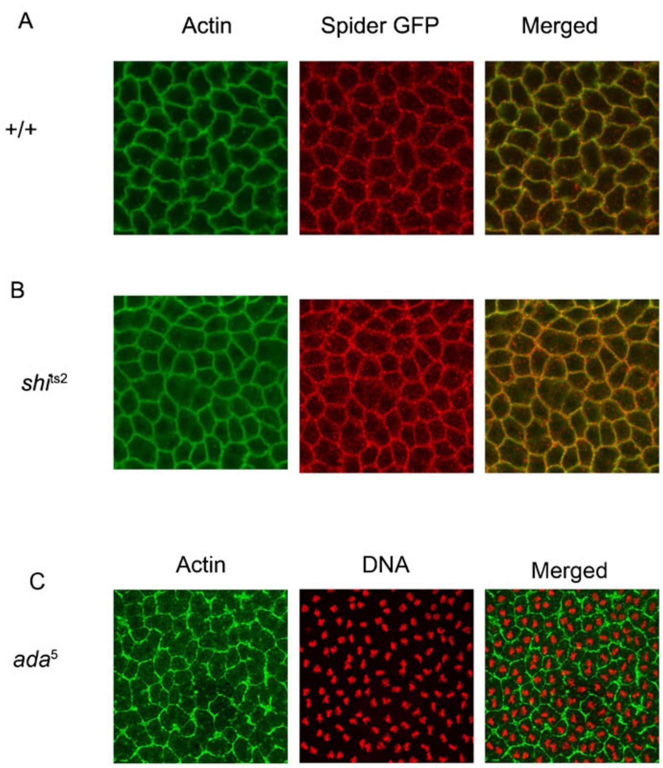

Fig. S2. Membrane and actin distribution in *shi*^{ts2} and α-adaptin mutant embryos. (A,B) Actin and Spider-GFP completely colocalized in control (+/+) and *shi*^{ts2} mutant embryos in metaphase of the syncytial cycle. (C) α-adaptin mutants showed a partial loss of metaphase furrow formation like dynamin mutants in the syncytial cycle of the *Drosophila* embryo.

Biology Open
